# The Lunacy Commission

**Published:** 1908-09-19

**Authors:** 


### The Lunacy Commission.

The sixty-second report of the Commissioners in Lunacy contains several points of interest. After giving the excess of patients over the number of last year, the Report shows that this increase is considerably below the rate of increase for the last ten or the last five years. This hopeful aspect seems at first sight to be negatived by the fact that since 1859 the rise in number of certified insane is from 36,762 to 126,084, or 243 per cent., while the estimated increase in the general population in the same period is 79.6 per cent. It must, however, be borne in mind that there is far more attention paid nowadays to mental aberration than was the case fifty years ago. Differentiations have taken place, and the connotation of insanity has certainly been extended. Again, the Report states that no small proportion of those who become insane do recover and resume their place in the world without again requiring to be segregated from the rest of mankind. This seems to suggest that the strain of modern life is at present more apt to throw a man suddenly off his balance, without permanently affecting him. If we are to believe the theories on heredity propounded by Dr. Archdall Reid, we shall look upon the fact that the rise in insanity has not proceeded with regularity as indication that the race is acquiring immunity in some respects. Certain families are able possibly to withstand an attack to which their ancestors would have succumbed.

The problem of heredity in this case is one of grave importance, and affects the family very closely. A doctor is often asked whether a man may marry in whose family directly or collaterally are insane persons. Probably, if a careful scouting were made, it would be found that there is no family in England that does not possess some "weak-minded" member. Moreover, the Commissioners declare that three-fourths of the persons annually certified to be insane are suffering from their first attack. This allows one to hope that treatment is efficacious, and that hereditary insanity is really less frequent than is thought. There must naturally be a residue of those who are readmitted and become incurable. Among this class should be numbered homicidal maniacs—namely the criminal insane, whose confinement must be permanent. It is difficult to fix an absolute line of demarcation, but it seems not too sanguine to hope that other means may be taken in dealing with this latter class. We have before suggested azoospermia, produced by the use of $x$-rays, and even more drastic measures might be economical.

The Commissioners insist upon the necessity of an institution which should provide adequately for the care of the patients immediately after their discharge from an asylum. This seems a most reasonable contention, seeing that it is probably owing to a return to the old environment that some of the patients suffer a recrudescence of their malady. Still more necessary is it that there should be some ultimate board of control, which should lay down with as much accuracy as is possible the scientific methods to be used both in recognising and dealing with patients. The next Report will contain a new list of the causes which are to be considered as potent in causing insanity. In some cases it may be demonstrated that there is an inherited tendency to brain disturbance, while in others there may be no apparent physical derangement by which the disease can be explained. On the whole, therefore the outlook is not without hope. The greatest difficulty which has to be overcome is that caused by the influence of the personal equation. It is not easy to obtain an impartial criterion. It is not at all certain that the habitual criminal ought not to be classed under the head of permanent lunatic. We do not refer to the homicide, but to the petty offender.

